# Ethnobotanical study of indigenous knowledge on medicinal plant use by traditional healers in Oshikoto region, Namibia

**DOI:** 10.1186/1746-4269-7-10

**Published:** 2011-03-09

**Authors:** Ahmad Cheikhyoussef, Martin Shapi, Kenneth Matengu, Hina Mu Ashekele

**Affiliations:** 1 Science, Technology and Innovation Division, Multidisciplinary Research Centre- University of Namibia, Private Bag 13301, Windhoek, Namibia; 2 Social Sciences Division, Multidisciplinary Research Centre-University of Namibia, Private Bag 13301, Windhoek, Namibia

## Abstract

**Background:**

The objective of this study was to establish a regional profile of the indigenous knowledge system (IKS) for medicinal plant use and cultural practices associated with the healing process of these plants by traditional healers in the Oshikoto region, Namibia.

**Methods:**

An ethnobotanical survey was undertaken to collect information from traditional healers during September and October 2008. Data was collected through the use of questionnaires and personal interviews during field trips in the ten constituencies of the Oshikoto region. A total of 47 respondents were interviewed with most of them aged 66 and above.

**Results:**

The traditional healers in Oshikoto region use 61 medicinal plant species that belong to 25 families for the treatment of various diseases and disorders with the highest number of species being used for mental diseases followed by skin infection and external injuries. Trees (28 species) were found to be the most used plants followed by herbs (15 species), shrubs (10 species) and climbers (4 species). The average of the informant consensus factor (F_IC_) value for all ailment categories was 0.75. High F_IC _values were obtained for *Pergularia daemia*, and *Tragia okanyua*, which were reported to treat weakness and dizziness problems, snake bite, swelling and cardiovascular problems indicating that these species traditionally used to treat these ailments are worth examining for bioactive compounds.

**Conclusions:**

The traditional healers in Oshikoto possess rich ethno-pharmacological knowledge. This study allows for identifying many high value medicinal plant species, indicating high potential for economic development through sustainable collection of these medicinal plants.

## Background

Traditional healers are found in most societies. They are often part of a local community, culture and tradition, and continue to have high social standing in many places, exerting influence on local health practices; it is therefore worthwhile to explore the possibilities of engaging them in primary health care and training them accordingly [1]. Most African people still rely heavily on traditional medicine; traditional healers are often the first and last line of defense against most diseases such as headaches, coughs, diarrhoea, wound healing and skin diseases [2]. One advantage in preferring traditional medicine is that traditional healers are found within a short distance, are familiar with the patient's culture and the environment and the costs associated with treatments are negligible [3]. They work on body and mind together to help cure an illness. Traditional medical knowledge of medicinal plants and their use by indigenous healers are not only useful for conservation of cultural traditions and biodiversity but also for community healthcare and drug development in the present and future [4]. Since the beginning of this Century, there has been an increasing interest in the study of medicinal plants and their traditional use in different parts of the world [5-8].

There are about 2400 traditional medical practitioners in Namibia who are registered with the National Eagle Traditional Healers Association (NETHA), but the actual number practicing could be higher [9]. Approximately 200 Southern African plant species are available in Windhoek, which are mainly imported by traditional healers from other countries. However, the number of local plants used is found to be high, primarily originating from Kavango, Caprivi and other northern regions in Namibia. The traditional medical practitioners reported that 53 plant species were in demand for medicinal trade in Windhoek [9]. Some of the traditional medical practitioners encountered came from the Democratic Republic of Congo, Malawi, Tanzania, Zambia and Zimbabwe [10]. One study reported on traditional healing in Katutura, Windhoek; but did not report details such as species name of the actual plants used. The study mainly concentrated on the traditional healing practices, beliefs, witchcraft and human behavior [11]. The database of plants that occur in the Oshikoto region supplied by National Herbarium of Namibia (WIND) [12] contains 753 species. Cross-checking this list with the SEPASAL (Survey of Economic Plants for Arid and Semi-Arid Lands) database (http://www.kew.org/sepasalweb/sepaweb) revealed that 274 (36.4%) of these plants are used for medicinal purposes [10].

The documentation of the traditional medicinal plants used by the traditional healers in Oshikoto region is limited compared to the extent of the variety of cultures and the diversity of plants of the region. Furthermore, the majority of these studies are focused only on interviewing traditional healers [13] and listing the plants for food and medicinal use [14] without any detailed ethnobotanical study on the medicinal plant in the region. The present study was conducted in Oshikoto region and to the best of our knowledge; this is the first report on the medicinal plant use by the local traditional healers. The purpose of this study is to assess traditional medicinal plant knowledge specifically with regard to the traditional healer's demographic characteristics such as: gender, age, source of income etc., and to document the knowledge and the uses of medicinal plants used by the traditional healers in Oshikoto region, which is a part of an initiative to document baseline data for future pharmacological and phytochemical studies.

## Methods

### Area

**Figure 1:**
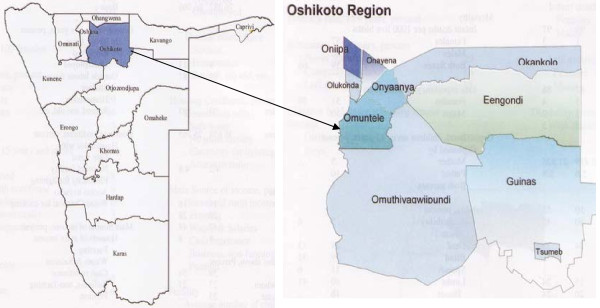
Map of the study area showing Namibia and Oshikoto region

The Oshikoto region was selected on the basis that it is inhabited by people from different ethnic groups: the San, Owambo, Damara/Nama, and Ova Herero. It was chosen as a pilot site because it could provide data useful for design of proper data collection instruments for a country-wide baseline study (Figure 1).

### Data Collection

Ethnobotanical knowledge and practice within any culture vary by geographical origin, residence, ethnicity, religion, age, and gender [15]. The biographic characteristics of the respondents in this study include: gender, age of respondents when first starting to practice traditional healing; occupational status and sources of income. This study mainly focused on the use of indigenous medicinal plants by traditional healers. No plant samples were collected. Forty seven respondents with a traditional knowledge of useful medicinal plants, mostly either native born or had been living in the region for long time were interviewed. The research team comprised of two groups; group A covered Oniipa, Onyaanya, Onayena, Olukonda and Omuntele constituencies during September 2008, while group B covered Okankolo, Engodi, Genius, Omuthiya and Tsumeb constituencies during October 2008. Each research team reported to the councilor of the constituency first before they could start with data collection. The councilor himself or somebody else recommended by the councilor led the research teams to the respective healers. Questionnaires designed to the respondents (traditional healers) about medicinal plants knowledge were mainly focused on common local name, knowledge about past and present use, mode of preparation, parts of the plants used, the methods of their preparation and administration, procurement method, place of collection and habitats, threats and conservation status, date/season of collection and types of treated diseases with these medicinal plants.

### Data analysis

#### Informant Consensus Factor (F_IC_)

The informant or respondent consensus factor (F_IC_) was calculated to estimate user variability of medicinal plants [16,17]. F_IC _values range from 0.00 to 1.00. High F_IC _values are obtained when only one or a few plant species are reported to be used by a high proportion of informants to treat a particular ailment, whereas low F_IC _values indicate that informants disagree over which plant to use [16]. High F_IC _values can thus be used to pinpoint particularly interesting species for the search of bioactive compounds [17]. F_IC _is calculated using the following formula [16,17]:

Where *N_ur _*is the number of individual plant use reports for a particular illness category, and *N_t _*is the total number of species used by all informants for this illness category.

### The fidelity level (FL)

The fidelity level (FL), the percentage of informants claiming the use of a certain plant for the same major purpose, was calculated for the most frequently reported diseases or ailments as following:

Where *Np *is the number of informants that claim a use of a plant species to treat a particular disease, and *N *is the number of informants that use the plants as a medicine to treat any given disease [6,18,19]. Prior to the calculation of FL, reported ailments were grouped into major disease categories following the approach of Heinrich et al. [16]. Generally; plants which are used in some repetitive fashion are more likely to be biologically active [20].

### Results and discussion

#### IK and Gender

Generally, traditional healing is a gender-based practice; although in some communities both men and women perform this practice [21]. It was found that among 47 respondents interviewed, 70% were women healers compared to only 30% of men. Women often consider traditional healers first for the treatment of infertility, whereas men's first option is frequent remarriage [22]. Problems related to women fertility are in many cases treated by the women healers though to lesser extent male healers also treat it. However, in some societies such as Kavango and Caprivi the situation is different of which men dominate the sector. The source of the medicinal plants knowledge is the main factor for the difference in knowledge of medicinal plants between women and men, and among respondents [19]. The women learn from their mothers or fathers through routine observations while their fathers' teach the man, in addition to routine observations, since the traditional knowledge in the family or community is passed from male parent to his first-born son [23].

### IK and Age

**Figure 2:**
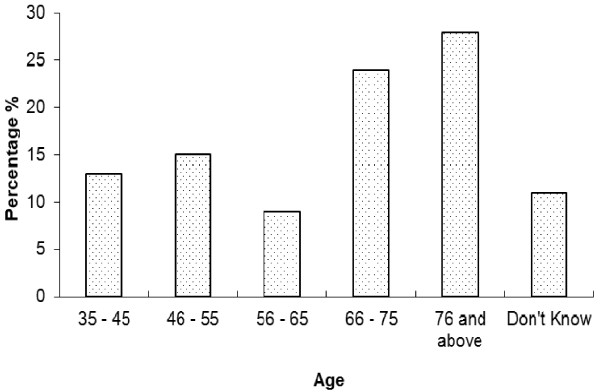
Percentage of healers by age category in Oshikoto, Namibia

**Figure 3:**
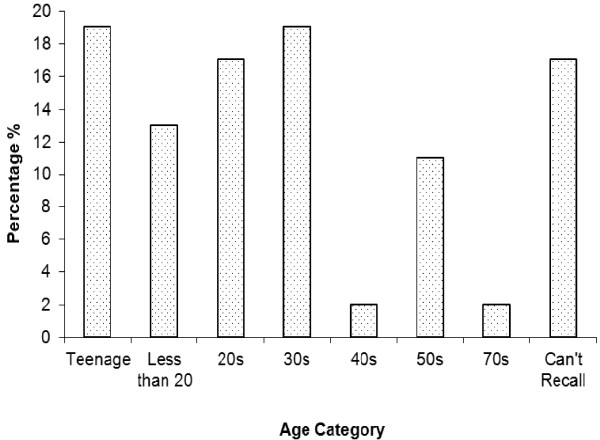
Age when the healers started healing in Oshikoto, Namibia

Indigenous knowledge (IK) is acquired through time in which it takes for the traditional healers to acquire the indigenous knowledge [21]. Most of the people who were known to have substantive knowledge and were practicing, were mostly older than 66 years of age (Figure 2). Bearing in mind the fact that IKS is passed on from one generation to another over time, Figure 3 indicates that less than 20% of the respondents became healers when they were teenagers and less than 20% when they were in their thirties.

### IK, occupation and source of income

**Figure 4:**
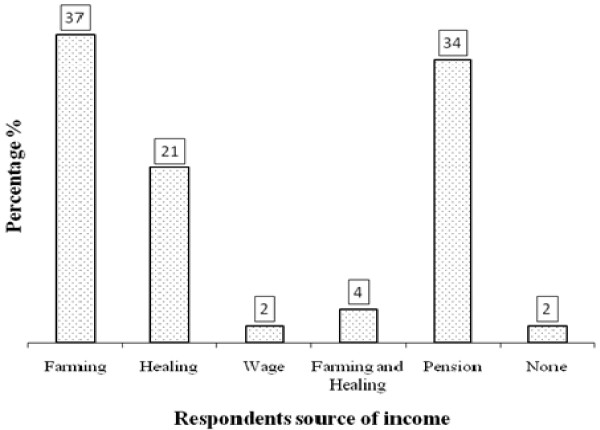
Percentage of respondents' source of income in Oshikoto, Namibia

The traditional healing is widely acknowledged as an occupation in Namibia. This study indicates that 58% of the respondents regarded themselves as subsistence farmers. Although these particular respondents use medicinal plants to treat ailments, they do not necessarily see themselves as practitioners of a profession/occupation. Twenty eight percent confirmed that they are traditional medicinal practitioners, while four and two percent reported to be pensioners and headman, respectively. It is evident that 37% and 34% of the respondents indicated that farming and pension, respectively; are their main sources of income. However, slightly more than 20% indicated that use of medicinal plants is their main source of income (Figure 4). This shows that a certain proportion of people do make a living from using medicinal plants hence the need to promote it. According to the World Health Organization (WHO), an estimated 3.5 billion people in the developing world depend on medicinal plants as part of their primary health care [24]. Also according to the WHO; 80% of the world's people depend on traditional medicine for their primary healthcare needs [1]. There are considerable economic benefits in the development of indigenous medicines and in the use of medicinal plants for the treatment of various diseases [25].

### IK and mentorships

**Figure 5:**
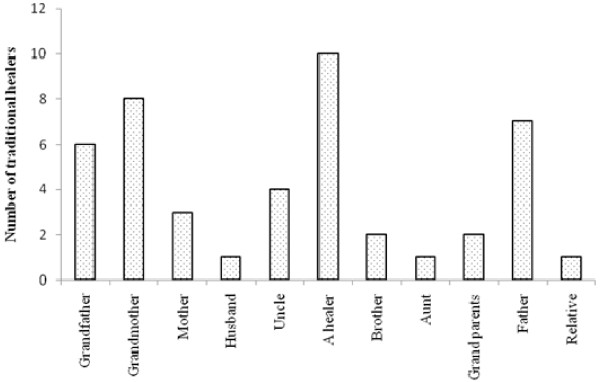
various people who played a mentorship role in passing IKS in Oshikoto, Namibia

Generally in many cases skills and knowledge are usually transferred from grandparents to their grandchildren [21]. It is common practice for grandchildren to accompany their grandparents during the medicinal plants collection and during the processes of treating patients. Through these interactions, these children become interested and may be motivated to also practice this profession. Nevertheless, in this study Figure 5 shows that a big number of the respondents indicated that they became healers through another healer, an indication that some form of initiation takes place. The information collected does not detail how exactly this interaction is initiated and the form it takes. Moreover, we do not know what circumstances may have persuaded the healer and the recruited healer to interact. However, 8 respondents indicated that they became healers because their grandmothers were healers, while 6 respondents said that they were healers because their grandfathers were healers also. Seven respondents claimed to have become healers through their grandparents without specifying whether it was their grandmother or grandfather. If one combined the number of the respondents who indicated that they became healers through grandfather, grandmother and grandparents mentorship, then it qualifies the African assertion that usually, traditional healing is mainly passed from grandparents to grandchildren because of the close interaction between grandparents and grandchildren as compared to his or her own children.

### Rituals for Practicing as Healer

It is generally believed that to become an acclaimed healer one has to go through the traditional ritual that will allow you to practice as a qualified healer. This process is called *lusha *or *okutetwa *in Oshiwambo language [21]. The survey in this study shows that 75% of the respondents indicated that they went through that process (*lusha *or *okutetwa*) before they became traditional healers. This is a sign that signifies the importance of indigenous knowledge in the sense that the community tends to believe that once someone goes through that ritual she or he can be trusted as someone who knows his or her work well. Some applications noted in some areas of Namibia were not compatible with western medical tradition, as local traditional healers have a different understanding of how certain diseases are caused. This is largely because traditional healing is a very complex issue involving rituals and spiritual aspects, far more than simply the use of plants [11,26].

### Traditional Healing and Mentorship

**Figure 6:**
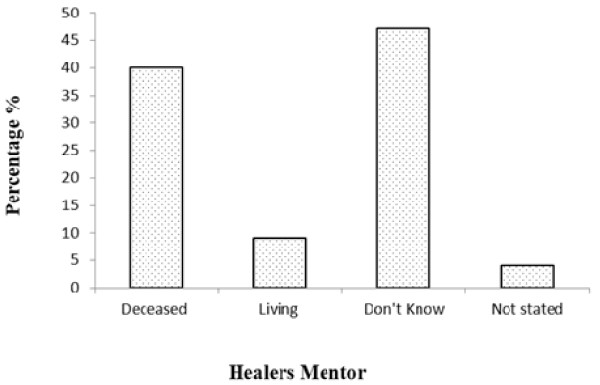
Healers knowledge about their mentor in Oshikoto, Namibia

In cases where people indicated that they were mentored, the study sought to establish whether the respondents knew the whereabouts of their mentors. The findings in Figure 6 show that over 45% of the respondents did not know where their mentors were and 38% indicated that their mentors had passed away long time ago. This is no surprise since the majority of the respondents were 66 years and above. Only less than 10% of the respondents indicated that their mentors were still alive. The low number of the respondent who indicated that their mentors were living, points to the importance of recording and documenting the IK on one hand and on the other hand, it is indicative of fading participation by the youth in IK. Thus, it can be suggested in the future studies that most of the respondents would not be younger than 30 years of age.

### Types of diseases treated

The traditional healing system in Oshikoto region is well established. Forty seven respondents reported 61 medicinal plants that are used to treat 43 types of ailments and diseases in both human and animal (Table 1). Several common diseases such as leg pains, back pains, chicken pox, ear infection, gonorrhoea, syphilis, stroke, diarrhoea, wound healing and skin diseases were reported to be among the illnesses that traditional healers can treat. This result is in agreement with Busia [27] who reported on the common ailments in Africa such as headaches or coughs are considered to be diseases with natural causes and hence their symptoms are treated at the household level. Various studies have reported on the indigenous use of medicinal plants in the treatment of oral diseases [28] wound healing and skin diseases [29] gastro-intestinal disorders [30]. Many of the remedies which are prescribed by traditional healers can be considered as general health tonics, used to treat weakness and unspecified ailments. Interestingly, there are specific mixtures of different medicinal plants that are often used to treat problems of the stomach, back, kidneys, bladder and other minor ailments [31]. The respondents reported that some types of ailments or diseases can be recovered after one day of treatment such as high fever, sun burns, and ear infection, on the other side some ailments take up to one year like: herpes and bilharzia (Table 1). Table 1 summarizes the ailments and IKS plant-based medicinal plants therapies in Oshikoto region.

**Table 1:** Medicinal plants used to cure various ailments in Oshikoto region, Namibia

Scientific name	Family	Local name	Ailment	Time of recovery
*Acacia ataxacantha*	Fabaceae	Mukoro	Headache	3 days
			Pneumonia	3-5 days
*Acacia mellifera*	Fabaceae	Okadilanghono	Snake bite	1-2 days
*Acanthosycios naudiniana*	Cucurbitaceae	Ruputwi	Mental illnesses	2 - 3 days, up to 16 months
			Inflammation	Vary
*Aloe littorals*	Asphodelaceae	Endombo	Bilharzia	1 year
*Asparagus nelsii*	Asparagaceae	Okawekamuthithi	Eczema	1 - 7 day
*Asparagus virgatus*	Asparagaceae	Runkwanana	Mental illnesses	2 - 3 days, up to 16 months
*Baphia massaiensis*	Fabaceae	Omulyambambi	Fertility/impotent	1 day
*Boscia albitrunca*	Capparaceae	Munkudi	Syphilis	4 days (depends on the severity)
			Chicken pox	2 days
*Clerodendrum ternatum*	Lamiaceae	Shanyu	Eczema	1 - 7 day
*Colophospermum mopane*	Fabaceae	Omusati	Swollen legs	One week
*Combretum collinum*	Combretaceae	Mububu	Coughing	1 week
			Panaritium	1 week
*Combretum imberbe*	Combretaceae	Omukuku	Gonorrhea	2 -7days
			Fertility/impotent	1 day
			Male dysfunction	1-4 day
*Croton gratissimus*	Euphorbiaceae	Ongonyo	Tetanus	2 - 3 days
			Lightening burn	One day
*Cyperus papyrus*	Cyperaceae	Onziku	Giving birth	1 day
*Datura stramonium*	Solanaceae	Egwitha	Mental illnesses	2 - 3 days, up to 16 months
*Dicoma tomentosa*	Asteraceae	Okalupulupu	Intestine cram	1 -3 days, 4 - 5 Months
*Diospyros lycoides*	Ebenaceae	Oshimumu	Bleeding	1 - 4 days
			Higher fever	1 day
*Diospyros mespiliformis*	Ebenaceae	Omwandi	Male dysfunction	1- 4 day
*Dregia macrantha*	Apocynaceae	Ondhingulula	Male dysfunction	1- 4 day
			Easing birth	Few hours
			Mental illnesses	2 - 3 days, up to 16 months
*Erythropheum africanum*	Erythroxylaceae	Mupako	Gonorrhea	2 -7days
			Mental illnesses	2 - 3 days, up to 16 months
*Euclea divinorum*	Ebenaceae	Omudime	Bleeding	1 - 4 days
*Euphorbia monteiroi*	Euphorbiaceae	Oshilalanamawadi	Mental illnesses	2 - 3 days, up to 16 months
*Ficus sycomorus*	Moraceae	Omukwiyu	Dermatitis	1 month
*Gloriosa virescens*	Colchicaceae	Ompilu	Intestine cram	1 -3 days, 4 - 5 Months
*Harpagophytum procumbens*	Pedaliaceae	Ekakata	Epilepsy	1 month
*Harpagophytum zeyheri*	Pedaliaceae	Ekakata	Epilepsy	1 month
*Helinus intergrifolius*	Rhamnaceae	Murora	Leg pains	2 -7days
			Stroke	2 weeks, 4 -6 months
*Helinus spartoides*	Rhamnaceae	Omutiwoheva	Leg pains	2 -7days
			Stroke	2 weeks, 4 -6 months
*Heliotropium *sp.	Boraginaceae	Ohanauni	Inflammation	Vary
*Kleinia longiflora*	Asteraceae	Elangelamwali	Mental illnesses	2 - 3 days, up to 16 months
			Colloid	Vary
*Kohautia angolensis*	Rubiaceae	Omutingulula	Intestine cram	1 -3 days, 4 - 5 Months
*Lonchocarpus nelsii*	Fabaceae	Mupanda	Back pains	2 days
*Peltophorum africanum*	Fabaceae	Omupalala	Leg pains	2 -7days
*Pergularia daemia*	Apocynaceae	Eriko	Mental illnesses	2 - 3 days, up to 16 months
*Plumbago zeylanica*	Plumbaginaceae	Okadimeti	Bleeding	1 - 4 days
*Pterocarpus angolensis *	Fabaceae	Uguva	Coughing	1 week
			Leg pains	2 -7days
			Bleeding	1 - 4 days
*Rhigozum brevispinosum*	Bignoniaceae	Ngandu	Syphilis	4 days (depends on the severity)
*Ricinus communis*	Euphorbiaceae	Olumono	Epilepsy	1 month
*Securidaca longepedunculata*	Polygalaceae	Omudhiku	Stroke	2 weeks, 4 -6 months
*Senna italic*	Fabaceae	Okatundangu	Abscess	depends
*Spirostachys africana*	Euphorbiaceae	Omuhongo	Leg pains	2 -7days
*Strychnos cocculoides*	Loganiaceae	Maguni	Culture bound syndrome	2-3 Days
*Swartzia madagascariensis*	Fabaceae	Omukonati	Bleeding	1 - 4 days
*Terminalia sericea*	Combretaceae	Omugolo	Leg pains	2 -7days
*Tragia okanyua*	Euphorbiaceae	Okalilifa	Generalised oedema	4 - 5 months
Unidentified		Munzwe	Ear infection	1 day
Unidentified		Xhangau	Diarrhoea	2 - 3 days
Unidentified		!hare	Abdomen pain	2 days
Unidentified		Guturoa	Worms	2 - 3 days
Unidentified		Okayekaathithi	Meningitis	Vary
Unidentified		Oshihangena	Barren	Vary
Unidentified		Onzingankelo	High Blood Pressure	Vary
Unidentified		okasisiti	Fatigue	4-5 days
Unidentified		Oshihangena	Easing birth	Few hours
*Vangueria infausta*	Rubiaceae	Oshimbu	Dermatitis	1 month
*Vigna dinteri*	Fabaceae	Muyimbo	Herpes	1 year
*Ximenia americanum*	Olacaceae	Kakukuru	Scoliosis	1 week
			Culture bound syndrome	2-4 Days
			Gonorrhea	2 -7days
*Ximenia caffra*	Olacaceae	Ompeke	Fertility/impotent	1 day
			Culture bound syndrome	2-5 Days
			Gonorrhea	2 -7days
*Ziziphus mucronata*	Rhamnaceae	Mukekete	Gonorrhea	2 -7days

### Plant parts used and mode of preparation

**Figure 7:**
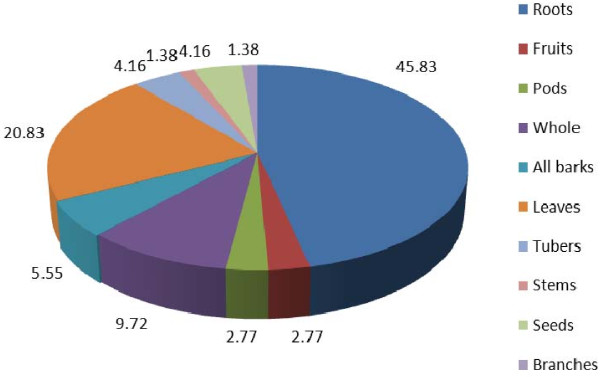
Percentage of medicinal plant parts used by the traditional healers

Different parts of medicinal plants are used as medicine by the traditional healers (Figure 7). Among the different plant parts, the roots are most frequently used for the treatment of diseases followed by leaves, whole plant parts, barks, tubers, seeds, fruits, pods, and stems. This result is in agreement with studies of Leffers [26] who presents and describes 238 plant species occurring in the eastern Tsumkwe Constituency, Nyae Nyae Conservancy in Namibia between 1999 and 2002 by one of the oldest and most fascinating cultures on the African continent, the Jul'hoansi. They use a wide range of different plants and plant parts, but plant roots play a significantly important role [26]. The result is also in agreement with Teklehaymanot [19] who reported that the roots used in the preparations in both single and multiple treatment with 58% and 48% respectively. On the other hand; the results of this study are not in agreement with some other studies which had addressed that leaves was the most common used parts for the treatments [32,33].

**Figure 8:**
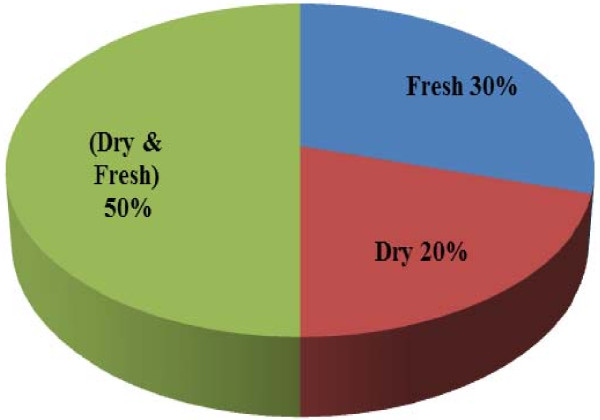
Status of use for medicinal plant parts

Regarding to the preparation method (fresh and dry) of used medicinal plants (Figure 8); it was found that 50% was used in both forms, then with 30% in fresh form and 20% for the dry form only. The traditional healers prescribed their treatment either on single plant use like the prescriptions for chicken pox, diarrhoea, ear infection and headache, or using a combination of different parts as it was prescribed for leg pains, bleeding, stroke and tetanus (Table 1). Similar results were also reported in various studies conducted in India [19,32,33]. This agreement could help for ethnopharmacological selection of plants for future phytochemical and pharmacological study [19,20].

### Life form and citied medicinal plants

**Figure 9:**
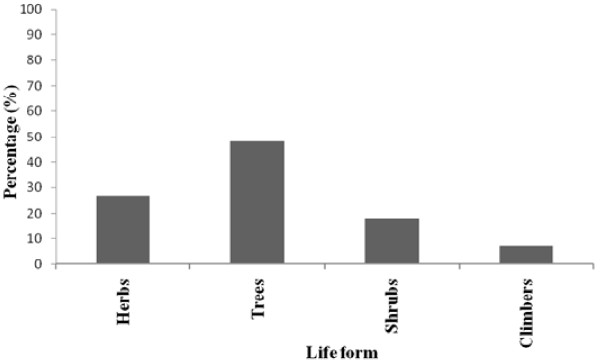
Percentage distributions of medicinal plant species according to the life form

**Figure 10:**
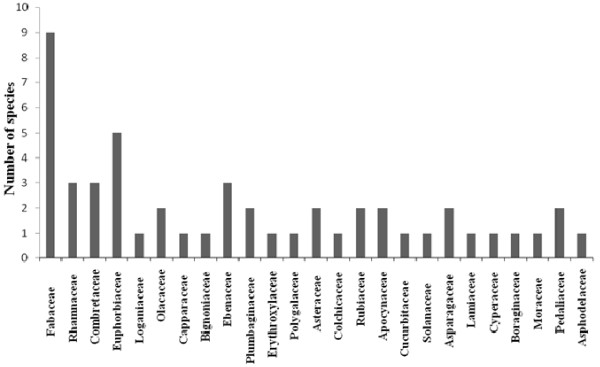
**Distribution of species in different plant families**.

Regarding to the life form of the reported medicinal plants by the respondents; the study showed that trees (28 species) were found to be the most used plants (Figure 9) followed by herbs (15 species), shrubs (10 species) and climbers (4 species) in descending order. The most dominant families in the study were Fabaceae (9 species), Euphorbiaceae (5 species), *Rhamnaceae, Combretaceae *and Ebenaceae (3 species). Other families with low number are listed below: Olacaceae, Plumbaginaceae, Asteraceae, Rubiaceae, Apocynaceae, Asparagaceae and Pedaliaceae (2 species), other families which represented with only one species are represented in Figure 10.

In this study; 47 respondents reported 61 medicinal plants species that belonged to 25 families. The most cited plants mentioned by the respondents are: *Pterocarpus angolensis, Ximenia americanum, Ximenia caffra *and *Combretum imberbe*. The total number of plant species used by the traditional healers normally will be diminishing in supply. Some plant species that are becoming more difficult to obtain include *Albizia brevifolia*, *Commiphora mollis*, *Commiphora glaucescens*, *Commiphora pyracanthoides*, *Elephantorhiza elephantina*, *Fockea angustifolia*, *Gardenia spatulifolia*, *Momordica balsamina*, and *Protea gaguedi*. *Protea gaguedi*, which occurred naturally along the Okavango River in Namibia, is practically extinct [14] due to frequent field fires and the over-exploitation of the roots for medicinal purposes; however these species are still available in neighbouring countries.

### Informant consensus

Table 2 shows the Informant consensus factor (F_IC_) for the different ailments treated by the traditional healers in Oshikoto region. The level of informants agreement was high for most ailment categories (mean FIC = 0.75) and total consensus (FIC = 1.00) was even obtained for weakness and dizziness problems, snake bite, swelling and Cardiovascular problems (Table 2). Cough and cold category, as well as infection and inflammation showed relatively low levels of consensus (FIC = 0.50). High F_IC _values indicating that the species traditionally used to treat these ailments are worth searching for bioactive compounds: *Pergularia daemia*, *Colophospermum mopane *and *Tragia okanyua*. These results are in agreement with Ignacimuthu et al [34] who reported that *Pergularia daemia *was used by tribes in Madurai district of Tamil Nadu, India for the treatment of headache. *Pergularia daemia *is found to be useful as anthelmintic, laxative, anti-pyretic, expectorant, and used in infantile diarrhoea [35]. The anti-inflammatory, anti-pyretic and analgesic activities of *Pergularia daemia *were reported also [36]. The plant was also found to possess anti-diabetic activity [37]. The proposed activities may be a result of the presence of flavonoid compounds; so the searching for bioactive compounds from this plant is very promising [35]. Fidelity level (FL) was calculated for medicinal plants which have been cited by four or more informants for being used against a given ailment. The following species: *Euclea divinorum*, *Erythropheum africanum*, *Baphia massaiensis*, *Kohautia angolensis*, *Asparagus nelsii*, *Clerodendrum ternatum *and *Ricinus communis *were the ones scoring the highest values (Table 3).

**Table 2:** Informant consensus factor (F_IC_) for different ailment categories

Ailment	Number of taxa (Nt)	Number of use reports (Nur)	F_IC_
Gastro-intestinal & Intestinal parasites	8	21	0.65
Fever and headache	2	5	0.75
Muscular-skeletal problems	4	10	0.66
Weakness and dizziness	1	2	1.00
Cuts and wounds	5	19	0.77
Cough and cold	2	3	0.50
Respiratory problems	2	4	0.66
Infectious & Transmitted diseases	6	15	0.64
Cardiovascular problems	1	2	1.00
Impotence	8	19	0.61
Birth problems	3	10	0.77
Mental diseases	6	26	0.80
Skin infection	7	25	0.75
Snake bite	1	2	1.00
Swelling	1	3	1.00
Neurologic diseases	8	21	0.65
Infection and Inflammation	5	9	0.50

**Table 3:** Fidelity level (FL) values of medicinal plants cited by four or more informants for being used against a given ailment

Medicinal plant	Local name	Therapeutic uses	Number of Informants	FL value (%)
*Euclea divinorum*	Omudime	Bleeding	5	100.00
*Erythropheum africanum*	Mupako	Mental illnesses	4	100.00
*Baphia massaiensis*	Omulyambambi	Fertility or impotence	4	100.00
*Kohautia angolensis*	Omutingulula	Intestine cram	8	100.00
*Asparagus nelsii*	Okawekamuthithi	Eczema	8	100.00
*Clerodendrum ternatum*	Shanyu	Eczema	5	100.00
*Ricinus communis*	Olumono	Epilepsy	4	100.00
Unidentified	*Xhangau*	Diarrhoea	4	100.00
*Diospyros lycoides*	Oshimumu	Bleeding, Worms	6	83.33
*Boscia albitrunc*	Munkudi	Chicken pox, Syphilis	4	75.00
*Ximenia caffra*	Ompeke	Gonorrhea, Culture bound syndrome, Fertility	6	66.66
*Pergularia daemia*	Eriko	Mental illnesses, Fertility or impotent, Snake bite	6	66.66
*Dregia macrantha*	Ondhingulula	Mental illnesses, Fertility or impotent, Erectile dysfunction	8	62.50
*Croton gratissimus*	Ongonyo	Tetanus, Lightening burn	5	60.00
*Pterocarpus angolensis*	Uguva	Leg pain, Coughing, Bleeding	7	57.14
*Acacia ataxacantha*	Mukoro	Headache, Bleeding, Pneumonia	7	57.14
*Kleinia longiflora*	Elangelamwali	Mental illnesses, Fertility or impotent, Colloid	6	50.00

At present the different policy and legislative instruments in Namibia addressing intellectual property rights (IPR) which cover areas such as bio-trade of medicinal plants, plant genetic resources, access and benefit sharing (ABS) need harmonization. However, Namibia is a Signatory to Cites, which also lists a number of fauna and flora species to be protected, FAO Treaty on exchange of agricultural research. There exists a Patent Office under the Ministry of Trade and Industry, but the absence of a policy regarding IPR as well as limited capacity to deal with IPR issues is a concern. Namibia is a Signatory to the Convention on Biological Diversity (CBD) which includes among other the ABS Protocol, and this protocol was adopted in 2010 in Nagoya, Japan. In Namibia, the Ministry of Environment and Tourism is responsible for this protocol and they are also working on a draft policy for ABS in Namibia. According to Du Plessis; Namibia has had a draft bill on ABS since 2007, but because of technical reasons, the bill has not been finalized and enacted into law [38]. To conduct research and collect plants require a permit from the Ministry of Environment and Tourism. The Namibian Government has instituted the National Biodiversity Programme (NBF), the Indigenous Plant Task Team (IPTT), and the Interim Plant Bio-prospecting Council (IPBC), mandated by Cabinet to formulate policies and legislation to regulate the above mentioned issues [39]. More recently, an Industrial Property Protection Bill was drafted and is being discussed by the respective legislative house in the Namibian Parliament. The proposed Bill is silent on issues of Indigenous Knowledge. Nevertheless, Namibia remains a very active player on IKS the SADC level and is in the process of developing an IKS National policy. It is envisaged that the policy will strongly address issues related to IPR of indigenous people, provide clear guidelines and protocols and IK research and the economic utilization thereof. The collaborative research study between the Directorate of Research, Science and Technology in the Ministry of Education and the Multidisciplinary Research Centre at the University of Namibia, which started in 2008 on Indigenous Knowledge Systems (IKS), will contribute to the drafting of the policy to regulate and coordinate the IKS issues in particular related to Traditional Healing (TH) and the use of knowledge generated from Indigenous Plants in Namibia.

Resolving of these issues is still at a primary phase because of the lack for national legislation relating to indigenous knowledge on indigenous plants. A Research, Science and Technology Act on this protocol was passed in 2004 to govern research activities and institutions in Namibia.

## Conclusions

This study showed that the study area has plenty of medicinal plants to treat a wide spectrum of human ailments. Many people in the study areas of the Oshikoto region are still depending on medicinal plants, at least for the treatment of some simple diseases such as, cold, cough, fever, headache, poison bites, skin diseases and tooth infections. It is necessary to acquire and preserve this traditional system of medicine by proper documentation and identification of specimens. A nationwide database is in the process of being established by collecting all conceivable IKS plant-based nationwide medicinal therapeutic data as currently applied by traditional healers. This traditional knowledge on the indigenous uses of the medicinal plants could boost new innovations in the pharmaceutical industry and have many beneficial applications such as new medicinal trails for some diseases like: malaria, Tuberculosis and AIDS, of which will develop the health care sector in Namibia.

## Competing interests

The authors declare that they have no competing interests.

## Authors' contributions

MS had interviewed the traditional healers in Oshikoto region; he has analyzed and drafted the report on the medicinal plant use by traditional healers. KM designed the questionnaire, and reviewed the manuscript. HMA led the research project and also reviewed the manuscript. AC has written the article, organized the compiled research data and carried out the statistical analysis. All authors read and approved the final manuscript.
